# Acute Effects of Breakfast Fruits Meal Sequence and Postprandial Exercise on the Blood Glucose Level and DPP4 Activity among Type 2 Diabetes Mellitus Patients: A Pilot Study

**DOI:** 10.1155/2022/4875993

**Published:** 2022-09-27

**Authors:** Dono Indarto, Dwipajati Dwipajati, Paramasari Dirgahayu, Yohanes Cakrapradipta Wibowo, Yoga Mulia Pratama

**Affiliations:** ^1^Department of Physiology, Faculty of Medicine, Universitas Sebelas Maret, Jl. Ir. Sutami No. 36A, Surakarta, Jawa Tengah 57126, Indonesia; ^2^Program of Nutrition Sciences, Universitas Sebelas Maret, Jl. Ir. Sutami No. 36A, Surakarta, Jawa Tengah 57126, Indonesia; ^3^Biomedical Laboratory, Faculty of Medicine, Universitas Sebelas Maret, Jl. Ir. Sutami No. 36A, Surakarta, Jawa Tengah 57126, Indonesia; ^4^Department of Nutrition, Politeknik Kesehatan Kemenkes Malang, Jl. Besar Ijen No. 77C, Malang City, Jawa Timur 65119, Indonesia; ^5^Department of Parasitology, Faculty of Medicine, Universitas Sebelas Maret, Jl. Ir. Sutami No. 36A, Surakarta, Jawa Tengah 57126, Indonesia; ^6^Department of Internal Medicine, Faculty of Medicine, Jenderal Soedirman University, Jl. Profesor DR. HR Boenyamin No. 708, Banyumas, Jawa Tengah 53122, Indonesia

## Abstract

**Objectives:**

Type 2 diabetes mellitus (T2DM) is a major global public health issue. Diet and physical exercise are modifiable factors that influence the glycaemic status of patients with T2DM. We aimed to investigate the acute effects of breakfast fruits meal sequence and postprandial exercise on the blood glucose level and dipeptidyl peptidase 4 (DPP4) activity among type 2 diabetes mellitus patients.

**Methods:**

A randomized pilot study recruited patients with T2DM who attended two primary health care centres in Tasikmadu District, Karanganyar Regency, and Kartasura District, Sukoharjo Regency, Central Java, Indonesia, from July to October 2016. Eligible patients (4 men and 32 women) were randomly divided into four treatment groups. Venous blood samples were analyzed for fasting and one-hour postprandial blood glucose (FBG and 1 h PPG) levels and DPP4 activity. Blood glucose levels were measured using a routine hexokinase method, and serum DPP4 activity was determined spectrophotometrically after incubation with the Gly-Pro-p-nitroanilide substrate.

**Results:**

Fruits last meal decreased FBG level whilst fruits first meal did not significantly decrease 1 h PPG level. Both treatments had no acute effects on DPP4 activity but the addition of postprandial exercise helped lower DPP4 activity. Fruit last and first meals showed significant opposite effects on mean changes of FBG level (*p* < 0.05).

**Conclusions:**

This preliminary report of fruits meal sequence is potentially involved in acute regulation of blood glucose levels and that it might be independent of DPP4 activity in Indonesian patients with T2DM. Moreover, postprandial exercise may be an important intervention for T2DM through the mediation of DPP4 but has no acute effects on the regulation of blood glucose levels. Further studies are required to investigate whether or not different types of fruits and longer treatment intervals can affect blood glucose levels and DPP4 activity differently. This study also gives an insight into the feasibility of conducting food order modification with or without the combination of postprandial exercise in a primary health setting for our next studies.

## 1. Introduction

Type 2 diabetes mellitus (T2DM) remains a major public health problem and is estimated to afflict hundreds of millions of people worldwide. The trend has changed dramatically in recent decades and the number of patients with T2DM will increase significantly, reaching 578 million by 2030 [1]. Glycaemic control plays an important role in the prevention of T2DM complications. Elevated FBG and 2 h postprandial glucose levels are correlated with increased diabetic vascular complications [2]. Increased 1 h PPG levels are also associated with increased diabetic complications and mortality [3].

Dietary modification and physical exercise have been recommended as the first lines of T2DM management to minimise the risk of developing T2DM complications [4, 5]. For example, increased vegetable consumption decreases diabetes complications, such as diabetic retinopathy [6, 7]. Additionally, modification of meal sequence, such as vegetable consumption before carbohydrates caused a decrease in glycaemic parameters [8–11]. Meanwhile, fruit consumption is also predicted to improve glycaemic parameters in patients with T2DM because the fibre and antioxidant contents of fruits are similar to those of vegetables [12]. Unfortunately, the investigation of modified meal sequences involving fruits is still limited.

In general, recent evidence has indicated that regular physical activity strongly correlates with a reduction in T2DM development [13]. The American Diabetes Association recommends lifestyle changes, including physical exercise and activity, as an important part of glucose management for people with prediabetes and diabetes. Physical exercise improves insulin release and sensitivity in patients with T2DM [14] by upregulating the expression of glucose transporter type 4 (GLUT-4) [5].

General diet recommendations have not specifically defined on when fruit should be eaten [15, 16]. Additionally, previous studies of meal sequences on the impact of fruits on T2DM management are still limited. This indicates that meal sequences are generally neglected and focus only on the number of fruits that need to be consumed. Although the recommendation of physical exercise for T2DM is available [14], the study on the potential role of postprandial exercise which can be integrated into T2DM management, is also limited. Therefore, to fill this research gap, herein we aimed to compare different fruit meal sequences with or without a combination of postprandial exercise (PE) on blood glucose levels and DPP4 activity in Indonesian patients with T2DM.

## 2. Materials and Methods

### 2.1. Study Design, Area, and Period

This is a follow-up from two previous reports of the same preliminary study [17, 18]. We have previously reported that after 7 days of treatment, consuming fruit first as the meal (FF) significantly reduced fasting DPP4 activity in patients with T2DM compared to the consumption of fruit as the last meal (FL) [17]. However, we found no significant decrease in 30 min postprandial DPP4 activity after 7 days of treatment. In another report, we found that 2 min of physical exercise (jumping jacks) significantly decreased blood glucose levels in patients with diabetes [18]. Previously, it was also known that the physical exercise of jumping jack has health impacts through modifications of endothelial cell function and glucose control [19, 20].

Therefore, the current report included all of those groups with the addition of a new group who consumed FF, followed by 2 min of PE. We described new data from FBG and DPP4 of 0 and 60 minutes. However, due to the overlapping with the previous pilot study, the characteristic data of participants are partly in accordance with (and with permission to) the previous reports [17, 18].

In this present study, we conducted a randomized pilot study. The fruit-treatment study was performed at two primary health care centres in Tasikmadu District, Karanganyar Regency and Kartasura District, Sukoharjo Regency, Central Java, Indonesia, from July to October 2016. During this period, we were able to recruit 37 research participants but one participant from the FF group was excluded due to significantly incomplete data.

### 2.2. The Selection of Research Participants

The research participants were selected using the following criteria: diagnosed with T2DM, aged 45–65 years old, and able to ingest fresh fruits. We excluded pregnant women, patients who were taking DPP4 inhibitors (sitagliptin, vildagliptin, and similar drugs), and patients with chronic diseases such as heart, renal, and hepatic diseases.

### 2.3. Ethical Clearance

The study protocol was approved by the Research Ethical Committee, Faculty of Medicine, Universitas Sebelas Maret, Public Hospital Dr. Moewardi, Surakarta (No: 502/VI/HREC/2017). Informed consent was obtained from all selected research participants to draw peripheral venous blood and to recall their food consumption within 24 h using questionnaires. All data were kept confidential.

### 2.4. Protocol of the Study

Before starting the study, all participants received healthy nutrition education for T2DM. They had to consume an equal proportion of breakfast for the 7 days of treatment. On day 1 and 7, we provided breakfast, the list of meals, and fruits for the participants, such as bananas, oranges, and papaya. On days 1 and 7, participants consumed 50 g bananas whereas from days 2–6 participants consumed papaya or orange, which were equivalent to 50 g banana. Research participants consumed the meals and fruits based on their study groups in front of investigators on days 1 and 7. The composition of the meals consumed on day 2 to day 6 was equally restricted to a specific composition that represented food treatments on day 1 and day 7 during breakfast and was closely monitored by the investigators every day. A postprandial exercise of jumping jacks was performed for 2 min after having breakfast.

The food composition consisted of carbohydrates (mixed brown and white rice), protein and fat sources (tofu, egg, and meat), and mixed vegetables. The proportion of each macronutrient was determined according to the classical diet composition for diabetic patients: 55% carbohydrates, 25% fat, and 20% protein. Vegetable consumption was based on American Diabetes Recommendation [21].

The research participants were randomly divided into four groups: (i) consumed FL without 2 min postprandial exercise, (ii) consumed FF without 2 min postprandial exercise, (iii) consumed FL, followed by 2 min postprandial exercise (FL + PE), and (iv) consumed FF, followed by 2 min postprandial exercise (FF + PE), respectively. The protocol of the study was retrospectively registered in the ISRCTN registry on 13^th^ May 2022 with the trial registration number 13920339 and is publicly available at https://doi.org/10.1186/ISRCTN13920339.

### 2.5. Characteristics of Research Participants

Basic characteristics of research participants were obtained from their medical records at the two primary health cares. Parts of the characteristics (FL and FF) are in accordance and with the permission of our previous studies [17, 18]. The glucose control level was assessed by 2 h postprandial glucose level which is the best indicator for glucose control in the absence of HbA1C [22].

### 2.6. Nutritional Intake

To assess the effects of treatment, we measured the participants' daily intake of macro and micronutrients. The data were acquired from every research participant with regard to quantity, size, food type, composition, and food processing using a 24 h food recall questionnaire on three different days and converted into daily intake values of macro- and micronutrients using the free NutriSurvey software (https://www.nutrisurvey.de/), which has been translated in Indonesian.

### 2.7. Blood Glucose Level

Blood glucose levels were measured on day 1 and day 7 of the 7-day treatment. Venous blood samples were used to measure FBG and 1 h PPG levels, which were examined using a routine hexokinase method in the clinical laboratory of two primary health cares.

### 2.8. DPP4 Activity Assay

10 *μ*l serum blood samples were diluted in 40 *μ*l phosphate-buffered saline (PBS) pH 7.4 and were mixed with 50 *μ*l of 2 mM Gly-Pro p-nitroanilide substrate (Sigma-Aldrich, St. Louis, MO, USA) to reach a 1 mM final concentration. Once the samples were mixed completely with the substrate, the DPP4 activity was measured spectrophotometrically within 60 min for every 10 min interval at *λ* = 405 nm at 25°C. Finally, DPP4 activity was calculated using the Beer–Lambert formula [23], *A* = *ε*Cl, where *A* = absorbance, *ε* = *μ* molar extinction coefficient (9.45 liters·*μ*mol–1 cm^−1^ for pNA at 405 nm), *C* = concentration (*μ*mol·litre-1) and *l* = length of the light path.

### 2.9. Statistical Analysis

All numerical data are presented as mean ± standard deviation, and categorical data are presented as numbers and percentages. SPSS version 20.0, for Windows (SPSS, Inc., Chicago, IL, USA), was used to analyse the statistical significance of our data. Normality and homogeneity tests were performed before comparing the averages of blood glucose levels and DPP4 activity among the groups. A comparison of the basic characteristics of the research participants was performed using the chi-square test while the paired Student's *t* test was performed to analyse the time-dependent difference of the parametric data. The Wilcoxon test was used to analyse non-parametric data. An independent Student's *t* test was used to analyse the time-independent difference of the parametric data while the Mann–Whitney test was used for non-parametric data.

## 3. Results

### 3.1. Characteristics of Research Participants

From 36 selected research participants, Table 1 indicates their general characteristics, of which the FL and FF groups consisted of 10 and 8 T2DM patients, respectively. The mean ages of the four groups were relatively similar but the FL group had the youngest mean age (53.50 ± 7.28 years), compared to the treatment groups (55.88 ± 4.19; 54.11 ± 6.07 and 55.56 ± 3.54 years). Females were more dominant than males in all four groups (>85%). The mean duration of T2DM in FF (7.31 ± 3.43 years) and FF + PE (5.56 + 3.60 years) groups was longer than that of in FL (4.65 + 3.48 years) and FL + PE (3.39 + 3.71 years) groups but it was not significantly different. Based on anthropometric parameters, most research participants were overweight and obese (65% or more) except in the FF group (50% normal weight and 25% overweight or obese).

### 3.2. Nutritional Intakes of Research Participants

We measured the nutritional intake of every research participant using a food recall approach. We observed that pretreatment nutritional intake varied among the groups. However, after meal sequence treatment during breakfast, along with a standardized diet in two other daily meals, equal nutritional intake was achieved after 7 days among the four groups, especially in dietary carbohydrate and fibre intake, which were our main interests.

**Figure 1:**
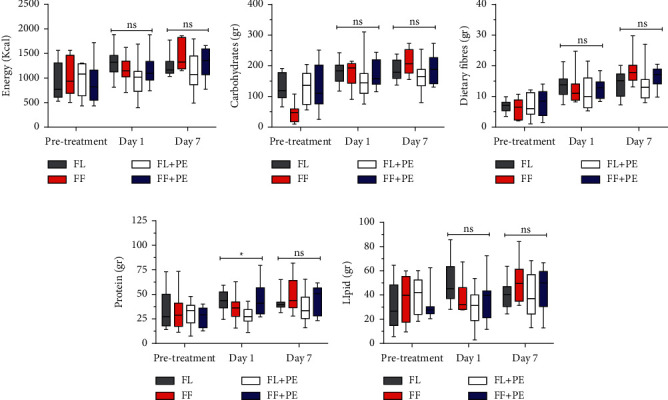
Nutritional intakes on pre-treatment and day 1 and day 7 of a 7-day course of treatment were as follows: (a) energy, (b) carbohydrates, (c) dietary fibre, (d) protein (gr), and (e) lipids (gr). Generally, no significant differences were found in macronutrients consumption on days 1 and 7 except protein consumption on day 1. ns = non-significant, ^*∗*^*p* < 0.05.

Within 7 days of treatment (Figure 1) there were no significant differences in the nutritional intake of energy, carbohydrates, fibres, proteins, and lipids among groups. However, protein intake on day 1 was significantly different between the groups.

### 3.3. Fruits Last Meal with or without Postprandial Exercise Decreased the FBG Level after 7 Days of Treatment

**Figure 2:**
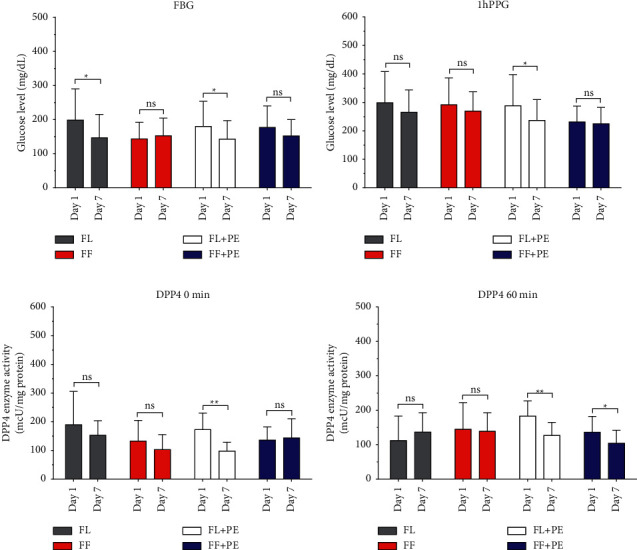
(a) Fasting blood glucose (FBG) level on day 1 and day 7 of a 7-day course of treatment; (b) one-hour postprandial glucose (1 h PPG) level on day 1 and day 7 of a 7-day course of treatment; (c) fasting DPP4 activity; and (d) 1 h DPP4 activity on day 1 and day 7. ns = non-significant, ^*∗*^*p* < 0.05.

Significant lowered FBG levels were observed in the FL (198.7 ± 91.64 vs. 146.9 ± 68.34; *p* = 0.45) and FL + PE (179.78 ± 74.19 vs. 143 ± 53.95; *p* = 0.032) groups after 7 days of treatment. However, we did not find any significant decrease in other groups (Figure 2(a)).

### 3.4. Fruits Last Meal and Directly Followed by 2 min Postprandial Exercise Decreased 1h PPG Levels after 7 Days of Treatment

In addition to lowering FBG levels, we found that FL + PE significantly lowered 1 h PPG levels after 7 days of treatment (Figure 2(b)). Meanwhile, no significant difference was observed between the FL and FL + PE groups in terms of 1 h PPG level (298.60 ± 109.53 vs. 287.89 ± 109.44; *p*=0.952) after 7 days of treatment.

### 3.5. Fasting DPP4 Activity Was Significantly Lower after 2 min of Postprandial Exercise for 7 Days of Treatment

We observed a significant difference in fasting DPP4 activity in the FL + PE group after 7 days of treatment. A significant difference in fasting DPP4 activity was also observed in the FL group vs. FL + PE group (152.8 ± 50.8 vs. 97.7 ± 30.9; *p*=0.009). Only the FL and FF groups showed the same trend on day 1 (Figure 2(c)).

### 3.6. The Addition of Postprandial Exercise Lowered DPP4 Activity

FL + PE and FF + PE groups had significantly decreased 1 h DPP4 activity after 7 days of treatment (Figure 2(d)). We also observed a significant decrease in DPP4 activity in the FL + PE group but not in the FF + PE group. In contrast, fasting and 1 h DPP4 activity were relatively stable in both FL and FF groups.

### 3.7. Fruits First Meal Showed Significant Opposite Effects on FBG Levels Compared to Fruits Last Meal

To further analyse the effects of each treatment in detail, we investigated the change of mean from each variable over 7 days of treatment (Table 2). We observed that FF significantly showed opposite effects on FBG level compared to FL (−51.80 ± 70.23 vs 9.00 ± 37.86; *p*=0.043). However, the addition of postprandial exercise did not enhance the FBG-lowering effect of FL and FF groups, as shown by FL vs FL + PE (−51.80 ± 70.23 vs −36.78 ± 42.42; *p*=0.327) and FF vs FF + PE (9.00 ± 37.86 vs −24.78 ± 47.09; *p*=0.124), respectively.

### 3.8. Fruits Last Meal Significantly Lowered Mean Changes of 1 h DPP4 Activity without Affecting 1 h Postprandial Glucose Level

After 7 days of treatment, a significant decrease of 1 h DPP4 activity was observed in the FL + PE group compared to the FL group (24.80 ± 93.28 vs −68.26 ± 42.11; *p*=0.022). However, we did not see the same decrease of 1 h PPG in all groups.

### 3.9. Postprandial Exercise Combined with Fruits First Meal Was Superior in Regulating DPP4 Activity

FF + PE group showed significant mean changes in both fasting (−75.08 ± 41.75 vs −7.17 ± 73.01; *p*=0.028) and 1 h DPP4 activity (−68.26 ± 42.11 vs −32.05 ± 36.32; *p*=0.047) compared to FL + PE group.

## 4. Discussion

Herein, this randomized pilot study showed the effects of a fruit-carbohydrate meal sequence combined with 2 min of postprandial exercise on blood glucose levels and DPP4 activity in patients with T2DM. Our study was conducted at Karanganyar and Sukoharjo Regencies, in Central Java, Indonesia, which is populated by approximately 800,000 residents. Karanganyar and Sukoharjo Regencies were located ca. 120 and 90 km, respectively, from Semarang, the largest capital city of Central Java. Thus, it reflected rural population areas in the southeastern part of Central Java, and most residents work in the agriculture sector. However, the overweight to obesity class of BMI was predominant in our groups of research participants, suggesting that the profile of our rural T2DM research participants was similar to that of urban Indonesian patients with T2DM [24].

One week before starting the study, we assessed the daily nutritional intake of our research participants and our findings indicated variation among the groups (Figure 1). When we started our breakfast treatment and educated them to follow T2DM standard diets in two other daily meals, we were able to achieve similar nutritional intakes after 7 days except for protein intake, which was similar to that on day 1. Therefore, we excluded the possibility that significant differences in nutritional intake among the groups influenced our results.

Our data indicated that the FBG levels in patients with T2DM who consumed FL without postprandial exercise were significantly reduced compared to other groups. Furthermore, a significant reduction in the FBG level was only observed in the FL + PE group after 7 days of treatment but not in the FF + PE group. Therefore, this suggests that consumption of fruit, irrespective of meal sequence and additional postprandial exercise, independently had a delayed effect on FBG levels.

The present research participants consumed bananas in their modified diet, which had high-fibre content [25]. Recent studies have shown that consuming high-fibre vegetables before carbohydrates can delay gastric emptying [8–11] and therefore inhibit carbohydrate absorption. Subsequently, it has a critical impact on the prevention of T2DM progression [26, 27].

When we investigated 1h PPG levels, only FL + PE showed a significant reduction after 7 days of treatment. However, no significant change in 1 h PPG after 7 days was observed in all groups. In addition, FL + PE showed a significant reduction in DPP4 activity compared to FL. Altogether, these findings suggest that postprandial exercise without meal sequence modification regulates DPP4 activity without affecting the 1 h PPG level.

The effect of physical exercise on the improvement of FBG levels has been reported before [28], in which physical exercise decreased FBG levels among participants with a higher baseline FBG level. Physical activity can control blood glucose levels by promoting glucose consumption in skeletal muscles [29] and glucose uptake by GLUT-4 in the muscle cell membrane [5]. A different research group found that physical exercise decreased DPP4 activity in patients with metabolic syndrome [30]. Based on our study, even though there was no additional reduction of DPP4 activity, FF + PE showed a higher reduction of both fasting and 1 h DPP4 activity compared to FL + PE, which indicates that the combination of fruit at last meal with physical exercise has a greater acute regulation on DPP4 activity.

This study gives a preliminary insight into the role of fruits meal sequences and postprandial physical exercise for T2DM management, which regulates an acute phase of glucose levels and is probably independent of DPP4 activity. From this pilot study, we will further investigate a randomized controlled trial (RCT) with a bigger sample and different choices of fruits. A separate RCT will be conducted to investigate the impact of postprandial physical exercise in T2DM patients.

Our study has some limitations due to sample size and basic characteristics, which were less reflective of the rural population and patients with T2DM as well as overweight or obesity. The 7-day period of our investigation was also relatively short. Therefore, we could not distinguish different responses that may have occurred to the same treatment of fruits order modification and physical exercise, based on ethnic groups or their food habits and conducting a research study in different areas in Indonesia.

## 5. Conclusions

This preliminary report of fruits meal sequence is potentially involved in acute regulation of blood glucose levels and it might be independent of DPP4 activity in Indonesian patients with T2DM. Moreover, postprandial exercise may be an important intervention for T2DM through the mediation of DPP4 but has no acute effects on the regulation of blood glucose levels. Further studies are required to investigate whether or not different types of fruits and longer treatment intervals can affect blood glucose levels and DPP4 activity differently. This study also gives an insight into the feasibility of conducting food order modification with or without a combination of postprandial exercise in a primary health setting for our next studies.

## Figures and Tables

**Table 1:** Basic characteristics of research participants from four different groups.

Characteristics	FL (*n* = 10)^¥^	FF (*n* = 8)^¥^	FL + PE (*n* = 9)	FF + PE (*n* = 9)	*p* value
Age (years)	53.50 ± 7.28	55.88 ± 4.19	54.11 ± 6.07	55.56 ± 3.54	ns^*θ*^

Duration of type 2 diabetes mellitus (years)	4.65 ± 3.48	7.31 ± 3.43	3.39 ± 3.71	5.56 ± 3.60	ns^*£*^

*Sex*					
Male	1 (10.00%)	1 (12.50%)	1 (11.11%)	1 (11.11%)	ns^*γ*^
Female	9 (90.00%)	7 (87.50%)	8 (88.89%)	8 (88.89%)	

*Anthropometrics* Height (cm) Weight (kg) Body mass index (BMI) (kg/m^2^) BMI categories Normal weight Overweight Obese	153.13 ± 7.21 62.99 ± 9.17 26.79 ± 2.73 2 (20.00%) 7 (70.00%) 1 (10.00%)	152.14 ± 5.17 58.74 ± 15.79 25.16 ± 5.64 4 (50.00%) 2 (25.00%) 2 (25.00%)	149.83 ± 3.86 58.03 ± 8.27 25.88 ± 3.77 3 (33.33%) 5 (55.56%) 1 (11.11%)	155.32 ± 8.49 66.60 ± 14.57 27.34 ± 3.61 2 (22.22%) 5 (55.56%) 2 (22.22%)	ns^*θ*^ ns^*£*^ ns^*£*^ ns^*γ*^
2 h PPG (mg/dL)	316.90 ± 154.60	316.50 ± 125.05	307.33 ± 150.46	267.67 ± 87.89	ns^*θ*^

2 h PPG, 2 h postprandial glucose. ^*¥*^Parts of this table are in accordance with and with permission from our previous studies [17, 18]. ^*θ*^statistical differences were performed by one-way anova. The ^*£*^Kruskall–Wallis test was used to determine the statistical significances, ^*γ*^statistics were conducted by using Fischer exact test.

**Table 2:** The mean changes of each treatment after a 7-day course of treatment.

	FL	FF	FL + PE	FF + PE	*p* value
FBG	−51.80 ± 70.23	9.00 ± 37.86	−36.78 ± 42.42	−24.78 ± 47.09	a, d
1 h PPG	−33.20 ± 90.86	−22.38 ± 68.43	−50.89 ± 57.46	−6.33 ± 51.04	ns
Fasting DPP4 activity	−36.63 ± 94.27	−29.85 ± 80.29	−75.08 ± 41.75	−7.17 ± 73.01	*f*
1 h DPP4 actvity	24.80 ± 93.28	−5.98 ± 74.01	−68.26 ± 42.11	−32.05 ± 36.32	*b*, *d*, *f*

^*a*^significant difference between FL and FF. ^*b*^significant difference between FL and FL + PE. ^*c*^significant difference between FL and FF + PE. ^*d*^significant difference between FF and FL + PE. ^*e*^significant difference between FF and FF + PE. ^*f*^significant difference between FL + PE and FF + PE. ns = non-significant.

## Data Availability

All raw and analyzed data are kept by Yohanes Cakrapradipta Wibowo and we will provide data on request by emailing him at wibowo.yohanec@gmail.com.
